# Impact of COVID-19 outbreak on ischemic stroke admissions and in-hospital mortality in North-West Spain

**DOI:** 10.1177/1747493020938301

**Published:** 2020-06-26

**Authors:** Herbert Tejada Meza, Álvaro Lambea Gil, Agustín Sancho Saldaña, Maite Martínez-Zabaleta, Patricia de la Riva Juez, Elena López-Cancio Martínez, María Castañón Apilánez, María Herrera Isasi, Juan Marta Enguita, Mercedes de Lera Alfonso, Juan F Arenillas, Jon Segurola Olaizola, Juan José Timiraos Fernández, Joaquín Sánchez, Mar Castellanos-Rodrigo, Alexia Roel, Ignacio Casado Menéndez, Mar Freijo, Alain Luna Rodriguez, Enrique Palacio Portilla, Yésica Jiménez López, Emilio Rodríguez Castro, Susana Arias Rivas, Javier Tejada García, Iria Beltrán Rodríguez, Francisco Julián-Villaverde, Maria Pilar Moreno García, José María Trejo-Gabriel-Galán, Ana Echavarría Iñiguez, Carlos Tejero Juste, Cristina Pérez Lázaro, Javier Marta Moreno, on behalf of the NORDICTUS Investigators

**Affiliations:** 1 Stroke Unit, Department of Neurology, 16488 Hospital Universitario Miguel Servet, Zaragoza, Spain; 2 Interventional Neuroradiology Unit, Department of Radiology, 16488 Hospital Universitario Miguel Servet, Zaragoza, Spain; 3 Instituto de Investigación Sanitaria de Aragón (IISAragón), Zaragoza, Spain; 4 Department of Neurology, Hospital Universitario Donostia-Donostia Ospitalea, San Sebastián, Spain; 5 Department of Neurology, 16474 Hospital Universitario Central de Asturias, Oviedo, Spain; 6 Department of Neurology, 83011 Complejo Hospitalario de Navarra, Pamplona, Spain; 7 Department of Neurology, Hospital Clínico Universitario de Valladolid, Valladolid, Spain; 8 Neurovascular Research Laboratory, Instituto de Biología y Genética Molecular, Universidad de Valladolid – Consejo Superior de Investigaciones Científicas, Madrid, Spain; 9 Stroke Unit, Department of Neurology, Hospital Universitario de Araba, Vitoria, Spain; 10 Department of Neurology, Complejo Hospitalario Universitario de Vigo, Vigo, Spain; 11 A Coruña Biomedical Research Institute, Department of Neurology, Complexo Hospitalario Universitario A Coruña, A Coruña, Spain; 12 Department of Neurology, Hospital Universitario de Cabueñes, Gijón, Spain; 13 Neurovascular group, Biocruces Bizkaia Health Research Institute, Osakidetza, Department of Neurology, Hospital Universitario Cruces, Barakaldo, Spain; 14 Department of Neurology, Hospital Universitario Marqués de Valdecilla, Santander, Spain; 15 Department of Neurology, Complejo Hospitalario Universitario de Santiago, Santiago de Compostela, Spain; 16 Department of Neurology, Complejo Asistencial Universitario de León, León, Spain; 17 Department of Neurology, 118003 Hospital San Pedro, La Rioja, Spain; 18 Department of Neurology, Complejo Asistencial Universitario de Burgos, Burgos, Spain; 19 Department of Neurology, Hospital Clínico Lozano Blesa, Zaragoza, Spain

**Keywords:** Stroke, stroke care, mortality, Spain, COVID-19, ischemic stroke

## Abstract

**Background and purpose:**

Spain has been one of the countries heavily stricken by COVID-19. But this epidemic has not affected all regions equally. We analyzed the impact of the COVID-19 pandemic on hospital stroke admissions and in-hospital mortality in tertiary referral hospitals from North-West Spain.

**Methods:**

Spanish multicenter retrospective observational study based on data from tertiary hospitals of the NORDICTUS network. We recorded the number of patients admitted for ischemic stroke between 30 December 2019 and 3 May 2020, the number of IVT and EVT procedures, and in-hospital mortality.

**Results:**

In the study period, 2737 patients were admitted with ischemic stroke. There was a decrease in the weekly mean admitted patients during the pandemic (124 vs. 173, p<0.001). In-hospital mortality of stroke patients increased significantly (9.9% vs. 6.5%, p = 0.003), but there were no differences in the proportion of IVT (17.3% vs. 16.1%, p = 0.405) or EVT (22% vs. 23%, p = 0.504).

**Conclusion:**

We found a decrease in the number of ischemic stroke admissions and an increase in in-hospital mortality during the COVID-19 epidemic in this large study from North-West Spain. There were regional changes within the network, not fully explained by the severity of the pandemic in different regions.

## Introduction

Since the first reported case in early December 2019, severe acute respiratory coronavirus 2 (SARS-CoV-2) infection, known as Coronavirus Disease 2019 (COVID-19), has become pandemic so rapidly that healthcare systems have been overwhelmed all around the world.^1–3^ In Spain, by 16th May, 231,350 cases and 27,650 deaths had been confirmed.^4^ Many extreme measures have been taken to contain the spread of the disease, such as locking down communities, which could have affected the optimal stroke care.

Spain has been one of the countries more heavily stricken by SARS-CoV-2, but this pandemic has not affected all regions equally. While some registered more than 3000 cases per day, others did not reach 120. In this context, there is general uncertainty in Spain regarding the real impact of the COVID-19 outbreak on hospital stroke admissions. Moreover, the necessary measures taken by hospitals, aiming to increase healthcare professionals' protection, and government measures to protect at risk patients from unnecessary admission, may have jeopardized the quality of care provided to stroke patients.

We aimed to analyze the impact of the COVID-19 pandemic outbreak on hospital ischemic stroke admissions as well as the use of reperfusion therapies and in-hospital mortality in tertiary referral hospitals from North-West Spain.

## Methods

### Study design

**Figure 1.**
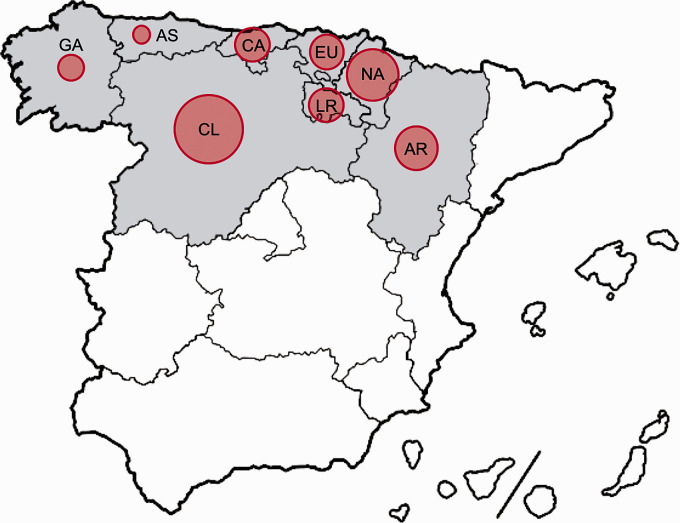
Map of Spain. Autonomous communities being part of NORDICTUS network are colored in gray. Red circles represent the percentage of seroprevalence in each region.

This Spanish multicenter retrospective observational study was based on the NORDICTUS network data. NORDICTUS is a research and innovation network in cerebrovascular diseases that brings together all public hospitals with stroke units in North-West Spain, with a global catchment area of 11.5 million inhabitants. According to its territorial division, the Spanish State is divided into 17 autonomous communities (AC) and two autonomous cities, both groups being the highest or first-order political and administrative division in the country. AC are divided into 50 total provinces and NORDICTUS covers 23, grouped in 8 AC represented in Figure 1, with the SARS-CoV-2 seroprevalence in each region by 14th May. Sixteen of the 18 referral centers of the network offered their data. During pandemic, none of the participating regions changed its prehospital ischemic stroke care.

Epidemiological data of COVID-19 cases were obtained from the Ministry of Health, Consumer Affairs and Social Welfare. It considers confirmed cases those who have a positive polymerase chain reaction for SARS-CoV-2. Due to a change in the counting system in the AC of Galicia, historical data from that administration were obtained from its regional Department of Health (Conselleria de Sanidade).^5,6^

### Study population

We reviewed the data from tertiary hospitals of the NORDICTUS network and recorded the number of patients admitted for ischemic stroke between 30 December 2019 and 3 May 2020. We grouped the cases in two periods, according to the setting of the state of emergency in Spain (14 March 2020) and considering the start of the COVID-19 period as the 11th week (W11) of 2020. We also recorded the number of intravenous (IVT) and endovascular treatments (EVT), as well as wake-up strokes or unknown-onset time. Finally, in-hospital mortality was recorded and analyzed as the key quality indicator of the stroke care process. Sixteen centers from eight different Spanish AC participated in this study.

### Statistical analysis

We used descriptive statistics to compare the incidence of stroke admissions before and after the setting of the state of emergency in Spain, expressed in strokes per week (W) and the differences between the other study variables (IVT, EVT, in-hospital mortality, and wake-up strokes or unknown-onset time) in those periods. Qualitative variables are described using counts and percentages, and continuous quantitative variables as means with standard deviation and medians with interquartile ranges when necessary. Comparisons between groups were made using chi-square tests for comparing categorical variables and the Student test or Mann-Whitney U test for continuous variables; p values < 0.05 were considered statistically significant. Statistical analysis was performed with SPSS Statistics 20.

### Ethics

The study was approved by the local Ethics Committee of each participating center. Treatment of every data obtained in the registry was done following the Spanish data protection law (Data Protection and Digital Rights Guarantee Act).

## Results

**Figure 2.**
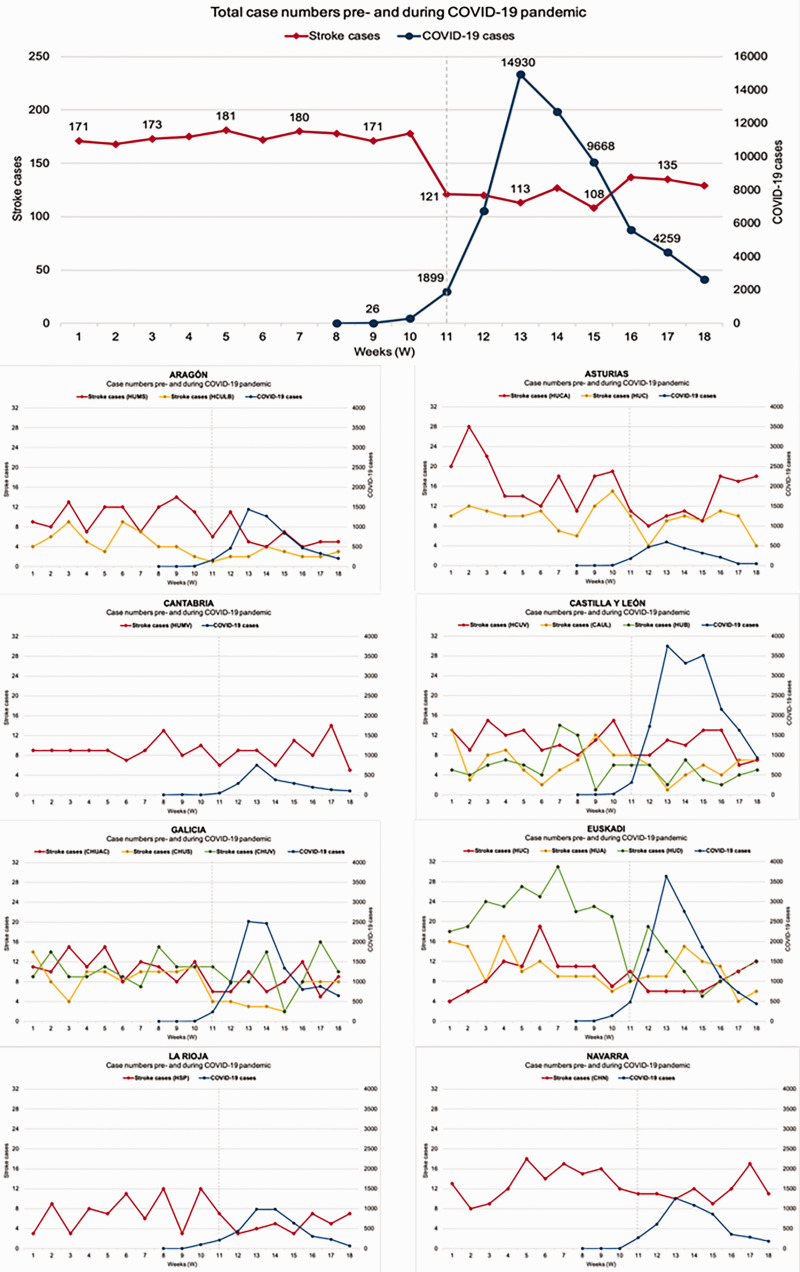
Case numbers pre- and during COVID-19 pandemic in NORDICTUS network regions, from 30 December 2019 (W1) to 3 May 2020 (W18).

In total, 2737 patients with ischemic stroke (male 1476, 53.5%; average age 73.5 years, SD ± 6.0) were attended to any of the hospitals participating in the study between 30 December 2019 and 3 May 2020. Table 1 shows global and specific results for each hospital and grouped by regions. Globally, there was a weekly average of 173 (IQR (interquartile range) 171.0–178.5) ischemic stroke admissions before the setting of the state of emergency against 124 (IQR 114.8–134.3) afterward (p < 0.001) (Table 1). This drop in stroke cases occurred progressively from week 11 (W11, 9–15th March), persisting over time despite the decrease in confirmed cases of COVID-19, but it did not occur homogeneously in each hospital; the reduction was only significant in 6 out of 16 centers (Table 1, Figure 2).

**Table 1.** NORDICTUS network data of acute ischemic stroke before (pre-COV) and after (COV) the setting of the state of emergency in Spain

		Strokes admissions per week (median, IQR)			IVT (n, %)			EVT (n, %)			UOS/>24 h (n, %)			Mortality (n, %)		
	n	pre-COV	COV	p	pre-COV	COV	p	pre-COV	COV	p	pre-COV	COV	p	pre-COV	COV	p
**Aragón**	224	14.5 (13.0–18.8)	7.5 (7.0–9.5)	**0.001**	39 (24.7)	15 (22.7)	0.449	34 (21.4)	17 (36.2)	0.390	49 (31.0)	17 (25.8)	0.268	12 (7.6)	7 (10.6)	0.461
HUMS	152	11.5 (7.8–12.3)	5.0 (4.3–6.8)	**0.002**	23 (21.9)	12 (25.5)	0.384	34 (21.4)	17 (36.2)	0.390	32 (30.5)	15 (31.9)	0.501	7 (6.7)	6 (12.8)	0.175
HCULB^a^	72	4.5 (3.8–7.5)	2.0 (2.0–3)	**0.004**	16 (30.2)	3 (15.8)	0.181	–	–	–	17 (31.1)	2 (10.5)	0.058	5 (9.4)	1 (5.3)	1.000
**Asturias**	449	27.5 (23.8–33.3)	21.0 (18.3–25.8)	**0.026**	25 (8.9)	16 (9.5)	0.486	53 (30.1)	25 (24.5)	0.194	90 (51.1)	65 (63.7)	**0.042**	–	–	–
HUCa	278	18.0 (13.5–20.5)	11.0 (9.3–17.8)	**0.025**	21 (11.9)	10 (9.8)	0.370	53 (30.1)	25 (24.5)	0.194	90 (51.1)	65 (63.7)	**0.042**	–	–	–
HUC^a,b^	171	10.5 (9.3–12.0)	9.5 (5.3–10.0)	0.076	4 (3.8)	6 (9.0)	0.146	–	–	–	–	–	–	–	–	–
**Cantabria** (HUMV)	160	9.0 (8.8–9.3)	8.5 (6.0–10.5)	0.377	8 (8.7)	5 (7.4)	0.499	15 (16.3)	22 (32.4)	**0.015**	30 (32.6)	26 (38.2)	0.284	4 (4.3)	6 (8.8)	0.204
**Castilla y León**	406	27.5 (22.–29.0)	19.5 (17.5–21.8)	**0.020**	41 (16.3)	20 (13.0)	0.226	56 (22.2)	42 (27.3)	0.151	96 (38.1)	54 (36.2)	0.397	14 (5.6)	14 (9.1)	0.123
HCUV	191	11.5 (9.0–13.5)	9.0 (7.3–12.5)	0.127	17 (14.8)	7 (9.2)	0.181	38 (33.0)	26 (34.2)	0.494	50 (43.5)	34 (44.7)	0.490	4 (3.5)	5 (6.6)	0.258
HUB	100	6.0 (4.0–8.3)	4.5 (2.3–6.0)	0.241	10 (15.4)	5 (14.3)	0.566	9 (13.8)	6 (17.1)	0.434	26 (40.0)	8 (26.7)	0.151	5 (7.7)	3 (8.6)	0.578
CAULE	115	7.5 (4.5–9.8)	6.0 (4.0–7.0)	0.245	14 (19.4)	8 (18.6)	0.558	9 (12.5)	10 (23.3)	0.108	20 (27.8)	12 (27.9)	0.576	5 (6.9)	6 (14.0)	0.181
**Euskadi**	668	42.5 (39.5–51.3)	28.0 (24.5–30.8)	**0.001**	57 (12.8)	31 (13.8)	0.402	75 (16.9)	46 (20.5)	0.147	51 (51.0)	17 (26.6)	**0.001**	29 (6.5)	18 (9.3)	0.146
HUD^b^	319	23.0 (20.5–25.5)	10.0 (8.0–13.5)	**0.001**	35 (15.0)	16 (18.6)	0.270	24 (10.3)	11 (12.8)	0.327	–	–	–	17 (7.3)	17 (19.8)	**0.001**
HUA^b^	185	9.5 (8.8–15.3)	9.0 (6.5–11.8)	0.322	17 (15.3)	13 (17.6)	0.416	11 (9.9)	5 (6.8)	0.320	–	–	–	7 (6.3)	10 (13.5)	0.082
HUC	164	110 (6.8–11.3)	7.0 (6.0–10.0)	0.206	5 (5.0)	2 (3.1)	0.439	40 (40)	30 (46.9)	0.240	51 (51.0)	17 (26.6)	**0.001**	5 (5.0)	4 (6.2)	0.494
**Galicia**	492	31.0 (28.8–34.5)	22.0 (18.8–27.8)	**0.002**	100 (31.9)	44 (24.6)	0.051	104 (33.2)	49 (27.4)	0.106	115 (36.7)	54 (30.2)	0.084	23 (7.3)	11 (6.1)	0.379
CHUAC	175	11.0 (9.5–12.8)	7.0 (6.0–9.8)	**0.014**	44 (38.9)	21 (33.9)	0.310	34 (30.1)	17 (27.4)	0.424	46 (40.7)	19 (30.6)	0.124	9 (8.0)	4 (6.5)	0.485
CHUS	135	10.0 (8.0–10.3)	4.0 (3.0–8.0)	**0.003**	33 (34.7)	11 (27.5)	0.270	23 (24.2)	7 (17.5)	0.268	50 (52.6)	24 (60)	0.276	10 (10.5)	5 (12.5)	0.474
CHUVI	182	10.0 (9.0–11.8)	9.0 (8.0–13.3)	0.529	23 (21.9)	12 (15.6)	0.190	47 (44.8)	25 (32.5)	0.063	19 (18.1)	11 (14.3)	0.317	4 (3.8)	2 (2.6)	0.496
**La Rioja** (HSP)^a,b^	115	7.5 (3.0–11.3)	4.0 (3.0–7.0)	0.092	8 (10.8)	8 (19.5)	0.197	–	–	–	–	–	–	1 (1.4)	2 (4.9)	0.289
**Navarra** (CHN)	227	13.5 (11.3–16.3)	11.0 (10.3–12)	0.152	29 (17.9)	19 (22.9)	0.222	10 (6.2)	5 (6.0)	0.603	63 (38.9)	35 (42.2)	0.359	14 (8.6)	12 (14.5)	0.162
**Total**	**2737**	173 (171.0–178.5)	124 (114.8–134.3)	**<0.001**	307 (17.3)	158 (16.1)	0.405	358 (22)	208 (23)	0.504	494 (39.4)	268 (37.7)	0.449	97 (6.5)	81 (9.9)	**0.003**

IVT: intravenous treatment; EVT: endovascular treatment; USO: unknown onsent stroke; > 24 h: more than 24 hours from stroke onset; HUMS: Hospital Universitario Miguel Servet; HCULB: Hospital Clínico Universitario Lozano Blesa; HUCA: Hospital Universitario Central de Austrias; HUCa: Hospital Universitario de Cabueñes; HUMV: Hospital Universitario Marqués de Valdecilla; HCUV: Hospital Clínico Universitario de Valladolid; HUB: Hospital Universitario de Burgos; CAULE: Complejo Asistencial Universitario de León; HUD: Hospital Universitario Donostia; HUA: Hospital Universitario de Álava; HUC: Hospital Universitario Cruces; CHUAC: Complejo Hospitalario Universitario de A Coruña; CHUS: Complejo Hospitalario Universitario de Santiago; CHUVI: Complejo Hospitalario Universitario de Vigo; HSP: Hospital San Pedro; CHN: Complejo Hospitalario de Navarra. Bold font indicates statistical significance.

a Stroke centers without Interventional Neurology Unit to perform endovascular treatment. These centers refer EVT to other NORDICTUS hospitals.

b Data of unknown onset or more than 24 h from stroke onset not available.

There were no differences in the proportion of ischemic stroke undergoing IVT (17.3% vs. 16.1%, p = 0.405) or EVT (22% vs. 23%, p = 0.504) during the pandemic or in the proportion of wake-up/unknown-onset strokes (39.4% vs. 39.1%, p = 0.887). In-hospital mortality of stroke patients increased significantly during the COVID-19 pandemic (6.5% vs. 9.9%, p = 0.003) (Table 1).

## Discussion

This study demonstrates a decrease in stroke admissions and an increase in stroke mortality during the COVID-19 pandemic across 16 centers within the NORDICTUS network including Aragón, Asturias, Cantabria, Castilla y León, Euskadi, Galicia, La Rioja, and Navarra in North-West Spain.

On 14 March 2020, the Government of Spain implemented extraordinary measures to control viral transmission, restricting free mobility over the entire country equally. This was reinforced from 31st March to 11th April, with essential workers the only ones allowed to leave their homes. These restrictions have been maintained until 4th May (W18). Since then, there has been a gradual return to normal by stages and which has varied between provinces. To date, Castilla y León is among the territories which maintain the most restrictive measures in Spain.

A decrease in hospital admissions for ischemic stroke in Europe is a situation that has already been referred to in different scientific media,^7–9^ but just described in two regional studies.^10,11^ This is the first study of which we are aware describing this phenomenon in hospitals from different regions throughout a wide coverage area in this continent. We found a decrease in the absolute number of ischemic strokes admissions, and although this was observed in all of the hospitals participating in the study, it only reached statistical significance in 6 out of 16 centers. If we group them by AC, the proportion increases so that only three out of eight territories (Cantabria, La Rioja, and Navarra; uni-provincial AC) did not show a significant decrease.

The magnitude of the decrease varied markedly between study centers. This varied from a drop in ischemic stroke cases of more than 50%, in three most affected hospitals compared to a drop of less than 10% drop in the three least affected hospitals (Table 1). In some cases, the variations were seen even despite being in the same AC and apparently with no correlation with the COVID-19 cases per week curves for each region (Figure 2). One example is the steep decline observed in hospitals from Asturias, which was less effected by COVID-19 cases than other regions.

Possible explanations for the decrease in ischemic stroke admissions have been suggested.^10–12^ These include changes in social behavior or attitude, minor non-disabling strokes staying at home, or admission to hospital isolation units where stroke might not be the major issue, or not enough attention being made to diagnose stroke. An argument against small strokes not being referred to hospital is our regional study from Aragón, one of the AC within the NORDICTUS network, in which we did not find a higher proportion of patients with higher NIHSS or lower ASPECTS compared to the pre-COVID period.^10^ Others speak about a possible real decrease in the incidence of strokes due to a reduction of risk factors such as air pollution.^9,13^

The increase in mortality, above the usual values in our area,^14^ could be explained by some of the previously described situations, or others such as fewer minor stroke admissions, increasing the proportion of severe ischemic strokes. It may also reflect an increase in stroke severity in patients with co-existent COVID-19 infection.^15^

Some authors have described a decrease in the number of IVT and EVT during this period.^11^ We also found this, but with no change in the proportion of treatments performed, similar to the findings of Zhao et al., who suggest the drop in the absolute number of IVT and EVT cases merely reflects the decline in stroke admissions.^12^

To the best of our knowledge, this study offers the biggest European sample to analyze the influence of COVID-19 pandemic in ischemic stroke admissions. We found a decrease in the number of ischemic stroke admissions and an increase in in-hospital mortality. Healthcare systems should be rapidly adapted to implement systems for COVID-19 care, but also to ensure the usual and effective stroke care despite system reorganizations. Since stroke is a life-threatening condition, it is important not to neglect the usual level of stroke care regardless of the difficult situation derived from the COVID-19 pandemic.

## Limitations

Our study has some limitations. The main limitation is inherent to its retrospective, observational nature. Besides, we did not investigate the incidence of virus infection among patients with stroke or whether it affected stroke outcomes. We did not obtain other stroke characteristics that could help to analyze the causes of the increasing mortality.

Second, although we analyzed data from sixteen hospitals with stroke units belonging to eight AC from the North of Spain, some ischemic strokes in the region are not admitted to these hospitals but instead to small hospitals without stroke units. However, due to their role as reference centers and as the hospitals with the highest volume of stroke patients in the area, we believe our data **give** a valid representation of the impact of COVID-19 in ischemic stroke over the whole region.

Finally, our results might not be extrapolated to other countries or regions with different stroke care protocols and different social and healthcare responses to the COVID-19 pandemic.
